# Effect of Acute Hypoxia on Cardiorespiratory Coherence in Male Runners

**DOI:** 10.3389/fphys.2020.00630

**Published:** 2020-06-30

**Authors:** Dmitriy Yu Uryumtsev, Valentina V. Gultyaeva, Margarita I. Zinchenko, Victor I. Baranov, Vladimir N. Melnikov, Natalia V. Balioz, Sergey G. Krivoschekov

**Affiliations:** Laboratory of Functional Reserves of Organism, Scientific Research Institute of Physiology and Basic Medicine, Novosibirsk, Russia

**Keywords:** athletes, hypoxia, cardiorespiratory coupling, squared coherence, cross-spectral analysis, heart rate, training

## Abstract

Understanding the mechanisms of oxygen supply regulation, which involves the respiratory and cardiovascular systems, during human adaptation to intense physical activity, accompanied by hypoxemia, is important for the management of a training process. The objectives of this study were to investigate the cardiorespiratory coherence (CRC) changes in the low-frequency band in response to hypoxic exposure and to verify a dependence of these changes upon sports qualification level in athletes. Twenty male runners aged 17–25 years were exposed to acute normobaric hypoxia (10% O_2_) for 10 min. Respiration, gas exchange, and heart rate were measured at baseline, during hypoxia, and after the exposure. To evaluate cardiorespiratory coupling, squared coherence was calculated based on 5-s averaged time series of heart and respiratory rhythms. Based on sports qualification level achieved over 4 years after the experimental testing, athletes were retrospectively divided into two groups, one high level (HLG, *n* = 10) and the other middle level (MLG, *n* = 10). No differences in anthropometric traits were observed between the groups. In the pooled group, acute hypoxia significantly increased CRC at frequencies 0.030–0.045 Hz and 0.075 Hz. In response to hypoxia, oxygen consumption decreased in HLG, and carbon dioxide production and ventilation increased in MLG. At 0.070–0.080 Hz frequencies in hypoxia, the CRC in HLG was higher than in MLG. Thus, highly qualified athletes enhance intersystem integration in response to hypoxia. This finding can be a physiological sign for the prognosis of qualification level in runners.

## Introduction

At rest and during sleep, the coupling of the cardiovascular and respiratory systems manifests as respiratory sinus arrhythmia (RSA) and cardiorespiratory phase synchronization (clustering of heartbeats within each respiratory cycle) (Moser et al., 1995; Kralemann et al., 2013; Bartsch et al., 2014). When metabolic demand increases, the phase regulatory system is inhibited (review: Hayano and Yuda, 2019). Stress tests from a battery of Ewing tests (handgrip static load and tilt test) have been shown to lead to the disappearance of intersystem phase synchronization (Sobiech et al., 2017). However, while moderate hypoxia does not change RSA, hypercapnic stress increases RSA amplitude in line with heart rate (HR) (Tzeng et al., 2007; Brown et al., 2014) and declines with increasing hypoxia in dogs (Yasuma and Hayano, 2000). Other work suggests that orthostatic challenge and cognitive load enhance the role of the baroreflex-mediated cardiorespiratory interaction (Krohova et al., 2018). Since the effect of baroreflex is demonstrated in the low-frequency (LF) band, it seems reasonable to suspect that stress induces a rise in cardiorespiratory coupling in a lower frequency range rather than in the range characterizing vagal tone.

Early data suggest that physical training changes patterns of cardiorespiratory interaction. Mlynczak and Krysztofiak (2019) used a Granger causality framework that parameterized cardiorespiratory causal link structures and directions to distinguish athletes from non-athletes at rest with 83% accuracy. Increased cardiorespiratory coordination after training has also been shown (Balagué et al., 2016). Training leads to a decrease in the number of principal components in cardiorespiratory response to acute physical exercise. For example, cardiovascular and respiratory responses to incremental cycling testing, which in most healthy individuals have two components, are simplified to one component after 6 weeks of aerobic and resistance training in more than half of the subjects (Balagué et al., 2016). *Prima facie* then, training appears to increase coordination among systems.

Since the integrating factor for the respiratory and cardiovascular anatomical systems is the oxygen supply, acute hypoxia provides an appropriate paradigm within which to assess how the integration between systems changes across physical training. We have previously demonstrated that the magnitude of the relationship between the hypoxic responses of the systems depends on the kind of sport and the athlete’s qualification level (Divert et al., 2015, 2017). In contrast to less experienced swimmers, for example, high-level swimmers show high intra-group correlations between cardiorespiratory indices in response to acute hypoxia and hypercapnia (Divert et al., 2017). Similarly, among ski racers, the highest results are achieved in the case of highly correlating chemoreflex responses of the respiratory and heart systems, assessed in hypoxic and hypercapnic tests (Divert et al., 2015). Limiting these data is the fact that findings were obtained by calculating correlations among time-averaged values. Temporal coupling of heart and respiratory waves under hypoxic stress in athletes with different qualification levels remains unknown.

In many high-performance sports contexts, results depend substantially upon oxygen supply and its regulation. Good coordination between the cardiovascular and respiratory systems under conditions of hypoxia can increase the physical capacity and physiological reserves of energy exchange. In particular, improvements in the mechanisms underlying the complex regulation of gas exchange, manifested in greater cardiorespiratory coherence (CRC) in the LF band, can be expected in high-level athletes. Commensurately, the objectives of the present study were to investigate CRC LF changes in response to hypoxic exposure and to verify a dependence of these changes upon sports qualification level in athletes.

## Materials and Methods

### Subjects

The study sample included 20 non-smoking middle-distance male runners aged 18–25 years. To complete the first objective, we analyzed the pooled group of athletes (*n* = 20). Based on sports qualification level achieved over 4 years after the hypoxic experimental testing, athletes were retrospectively divided into two groups, one high level (HLG, *n* = 10) and the other middle level (MLG, *n* = 10). We assigned those runners who had achieved the level of being a candidate for a master’s degree to HLG, according to the Russian sports classification scale, and those who had not to the MLG. Anthropometric characteristics did not differ between the groups (Table 1). To complete the second objective, we analyzed the dependence of the coherence changes in response to hypoxic exposure taking into account the HLG and MLG classification. All subjects provided written informed consent prior to participation. The study was approved by the Ethics Committee of the Scientific Research Institute of Physiology and Basic Medicine (Protocol No. 1 of 21.01.2016) and performed in accordance with the Declaration of Helsinki.

**TABLE 1:** Anthropometric characteristics of the subjects, Mean (SD).

Group	Height (cm)	Body weight (kg)	BMI (kg/m^2^)
Pooled	180.7 (5.7)	68.7 (8.0)	21.1 (1.6)
HLG	179.4 (6.7)	68.8 (8.9)	21.5 (1.8)
MLG	181.9 (4.6)	68.5 (7.5)	20.6 (1.4)

### Procedure

All investigations were performed in the morning by the same research assistant at an air temperature of 25°C in three functional states: at rest (baseline), during 10 min of breathing a hypoxic mixture with 10% O_2_ content through a face mask, and during the 10 min of breathing the ambient air (recovery). The specific duration of stages varied depending on the physical well-being of the subjects (range 8–10 min). The testing was conducted in a sitting position. The hypoxic mixture was prepared using an Armed 7F-3L (Russia) oxygen concentrator.

### Data Recording

A spiroergometric system, Oxycon Pro (Erich Jaeger, Germany), was used for recording the following respiratory parameters: breath rate (BR), carbon dioxide production (VCO2), oxygen consumption (VO2), and minute ventilation (VE). HR and blood oxygen saturation (SpO2) data were recorded by Pulse Oximeter BCI 3304 Autocorr (Smiths Medical, United States) and then automatically transferred to the Oxycon Pro. The Oxycon Pro software averaged data from the respiratory system and heartbeats and presented them at a maximum frequency of 0.2 Hz (period, 5 s).

### Data Analysis

Data analysis was performed using the STATISTICA10 software package (StatSoft). To meet the criterion of stationarity, we visually identified non-stationary areas occurring just after the onset of hypoxia and recovery period (1.5–2 min) and removed these parts from the analysis. We marked the corresponding items “remove trend” and “subtract the mean from the input series” in the STATISTICA10 software settings. To evaluate cardiorespiratory interaction, squared coherence was calculated based on Time Series Bivariate Fourier (Cross Spectrum) analysis of heart and respiratory rhythms (hereafter referred to as coherence). To estimate the spectral density of HR and BR, a Hamming window with a width of 5 points was used. Missing data (4.9%) were replaced by averaging nearby time points. For the group analysis, individual coherence values were superimposed on the frequency grid in 0.005 Hz increments by linear interpolation.

To analyze the effect of the hypoxia on dependent variables (SpO2, HR, BR, VCO2, VO2, VE, coherence values at different frequencies), two-way Repeated Measures Analysis of Variance (ANOVA) was conducted. The between-subjects factor was Group (two categories, HLG and MLG), and the within-subject factor was State (baseline/hypoxia). The main effects and interaction between factors (Group and State) were tested, and Fisher LSD *post hoc* tests were employed. The inter-group differences in anthropometric indices were assessed by the Student *t*-test. For conclusions, *p*-values < 0.05 were accepted to reject null hypotheses. However, *p* < 0.1 is also indicated to show a tendency of statistical difference. The calculated data are presented in figures as means and standard errors (Mean, SE) and in the tables as means and standard deviations (Mean, SD).

## Results

### Hypoxic Responses of Mean Values for Respiratory, Cardiac, and Gas Exchange Parameters

Overall, during hypoxia, oxygen saturation decreased by 21% and HR increased by 31%, but BR did not change significantly. Two-way repeated-measures ANOVA showed neither an effect of Group nor of Group × State on these parameters (Table 2). The reaction of gas exchange and pulmonary ventilation to hypoxia differed between groups. Oxygen consumption decreased only in HLG, and carbon dioxide production and minute ventilation increased only in MLG.

**TABLE 2:** Respiratory, cardiac, and gas exchange parameters at baseline and during hypoxia.

		Baseline		Hypoxia		ANOVA, *p*		
Parameter	Group	Mean	SD	Mean	SD	Group	State	Group × State
SpO_2_ (%)	Pooled	97.5	(1.1)	76.8	(6.1)		0.000	
	HLG	97.3	(0.9)	74.9	(7.6)	NS	0.000	NS
	MLG	97.8	(1.2)	78.7	(3.4)			
HR (beats × min^−1^)	Pooled	65.3	(9.0)	85.4	(11.4)		0.000	
	HLG	61.3	(8.9)	83.4	(15.2)	NS	0.000	NS
	MLG	69.2	(7.7)	87.4	(5.8)			
BR (breaths × min^−1^)	Pooled	13.5	(4.0)	14.0	(5.1)		NS	
	HLG	13.3	(4.1)	12.8	(3.4)	NS	NS	NS
	MLG	13.8	(4.1)	15.2	(6.2)			
VCO_2_ (mL × min^−1^)	Pooled	236.2	(40.2)	266.5	(76.4)		0.044	
	HLG	243.6	(43.5)	244.9	(83.9)	NS	0.028	0.034
	MLG	228.8	(37.4)	288.1	(65.0)*			
VO_2_ (mL × min^−1^)	Pooled	255.1	(35.8)	211.7	(59.0)		0.000	
	HLG	259.4	(35.2)	199.1	(68.7)*	NS	0.000	0.082
	MLG	250.8	(37.9)	224.3	(47.7)			
VE (L × min^−1^)	Pooled	10.3	(1.8)	12.2	(4.2)		0.026	
	HLG	10.4	(2,3)	10.8	(2.7)	NS	0.018	0.066
	MLG	10.2	(1,4)	13.6	(5.0)*			

SpO_2_, blood oxygen saturation; HR, heart rate; BR, breath rate; VCO_2_, carbon dioxide production; VO_2_, oxygen consumption; VE, ventilation; NS, non-significant; *significant difference between states at p < 0.01 (LSD post hoc) for the given group only.

### Cross-Spectral Analysis in the Pooled Group

**FIGURE 1:**
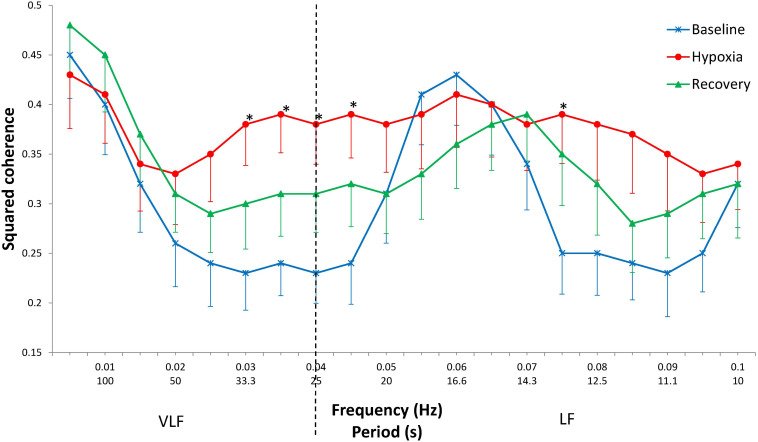
Squared coherence between cardiac and respiratory rhythms by frequency at baseline, during hypoxia, and after hypoxia (recovery). Mean ± SE. ^∗^Significant difference between baseline and hypoxia (*p* < 0.050).

At baseline, in the frequency range 0.025–0.045 Hz, a plateau zone is observed with a minimum coherence of approximately 0.23 and significantly higher coherence values at frequencies 0.050–0.070 Hz, with a group maximum value of 0.43 at a frequency of 0.060 Hz (LSD *post hoc*, *p* < 0.001). In hypoxia, the coherence values do not differ at all from the frequencies studied and are close to the above-mentioned maximum baseline level (Figure 1). At recovery, the coherence values are in an intermediate position between baseline and hypoxic values. Thus, the subsequent statistical analysis was performed for two states only (baseline and hypoxia).

The increase in coherence during hypoxia in comparison with the baseline values is mostly evident at frequencies 0.030–0.045 Hz (*p* < 0.050) and at a frequency of 0.075 Hz (*p* = 0.029). In the range 0.080–0.090 Hz, the coherence tended to increase (*p* = 0.053–0.066).

### Cross-Spectral Analysis in HLG and MLG

**FIGURE 2:**
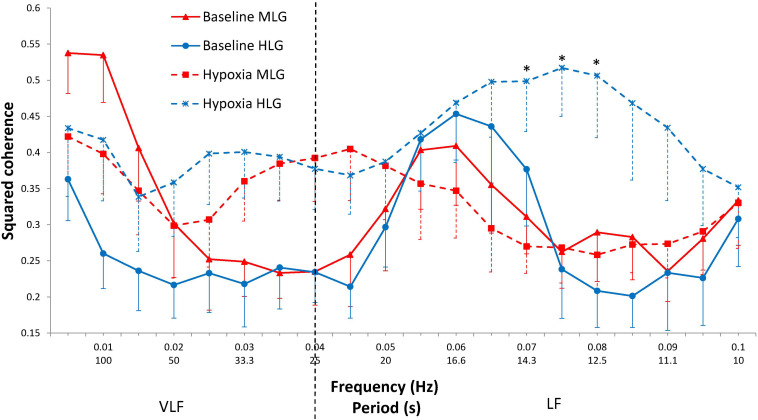
Squared coherence between cardiac and respiratory rhythms by frequency at baseline (solid lines) and during hypoxia (dashed lines) in MLG (red) and HLG (blue). Mean ± SE. *significant difference between groups for the hypoxic state (*p* < 0.030).

In response to hypoxia, the CRC in the 0.075–0.085 Hz range increases only in the HLG (interaction “Group × State,” *p* = 0.010–0.021) (Figure 2). In the range of 0.070–0.080 Hz during hypoxia, coherence was significantly higher in HLG than in MLG (*p* = 0.008–0.023). At frequencies of 0.030–0.045 Hz, the coherence gain during hypoxia does not differ significantly between groups (State distinct effect *p* = 0.001–0.016; Group effect and Group × State interaction effect did not reach a borderline level of significance). No effects were established for State, Group, or their interaction effects over frequencies 0.015–0.025 Hz and 0.050–0.065 Hz.

## Discussion

The present study establishes two novel facts. First, at rest, the peak CRC in the low–very LF band is around 0.060 Hz; hypoxia enhances the coupling at frequencies 0.030–0.045 Hz and 0.075 Hz. The second fact concerns differences related to the degree of sports training (and by inference, fitness): the hypoxic coherence at 0.070–0.080 Hz frequencies in high-level runners is significantly higher than in middle-level athletes.

The difference between highly trained versus moderately trained runners in response to hypoxia was also expressed in different dynamics of gas exchange and ventilation. In HLG, oxygen consumption decreased, but minute ventilation and carbon dioxide production did not change. In MLG, ventilation and CO_2_ production increased, whereas oxygen consumption did not change. Therefore, one can potentially conclude that high-level runners are characterized by reduced sensitivity to hypoxia. This allows them not to increase energy expenditure for the work of respiratory muscles. In the moderately trained group, however, there was an increase in ventilation, suggesting that the work of the muscles increased and, as a consequence, carbon dioxide production increased without total changes in oxygen consumption. It can be concluded that in more highly trained runners, there is a more economical mode of operation during hypoxia at physical rest. Increased CRC under hypoxic conditions probably plays a similar role: «Tuning and synchronization of rhythms saves energy» (Moser et al., 2006).

It has been shown previously that acute hypoxia causes an increase in sympathetic activity and an increase in sympatho-respiratory coupling in rodents (Dick et al., 2014). The enhanced respiratory-sympathetic coupling under these conditions is a result of synchronized activation of respiratory and sympathetic medullar neurons (Zoccal, 2015; Lindsey et al., 2018). In the present study, the growth of CRC at rest in normobaric hypoxia in humans is shown for the first time, and the prevailing frequencies at which it occurs were highlighted. The frequency component of HR variability, close to 0.076 Hz, is related to the baroreflex control of blood pressure (Kuusela et al., 2003). One can assume that an increase in CRC at this frequency is associated with an increase in the influence of baroreflex tuning in the heart and respiratory rhythms under the influence of hypoxia.

A physiological network is known to undergo topological transitions associated with rapid reorganization of interactions on time scales of several minutes (Bashan et al., 2012). It seems likely that higher coherence values during hypoxic stress in the more highly trained group are associated with a higher rate of network rearrangement. The 10-min hypoxia was sufficient for the athletes from this group to form new between-systems interactions, in contrast to less highly trained runners. Otherwise, one can assume that higher-level runners have developed more precisely coordinated responses to the exposure.

Mlynczak and Krysztofiak (2018, 2019) have studied cardiorespiratory links in sportsmen in normoxic conditions at rest by various mathematical non-linear methods. In the latest research, the authors were able to distinguish athletes from non-athletes with an accuracy of 83% on average. Platiša et al. (2019) have demonstrated modified neural control of heart-rate behavior in athletes compared with untrained subjects. In our study, runners differ in normoxia only in the qualification level, but we were able to demonstrate differences between moderately and highly trained runners in different reactions to hypoxic stress, reflected in greater coherence at frequencies of 0.07–0.08 Hz. It may be that greater training is reflected not only in changes in the mean values of cardiorespiratory parameters but also in alterations to the mechanisms that provide optimal (precise) settings of chemoreceptor reactions to developing hypoxemia. The less highly trained athletes may have cardiovascular and respiratory values similar to those in higher-skilled athletes at baseline, but the former may demonstrate less cardiorespiratory coupling at certain frequencies under conditions of hypoxia.

It has been shown earlier in our laboratory (Divert et al., 2015) that cardiorespiratory reactions to hypoxic and hypercapnic tests divide athletes into the clusters depending on the type of sports performance they are specialized in. This finding has initiated subsequent studies that led to the conclusion of a close, mutually substitutional relationship between respiratory and cardiovascular systems under hypoxic conditions (Melnikov et al., 2017). For example, it has been shown that the parameters of the training process and the features of the respiration pattern that appear as a consequence of training modulate the sensitivity of brain structures to hypoxia as reflected in changes in the EEG α-rhythm under conditions of hypoxia (Balioz and Krivoshchekov, 2012).

The results of the present study suggest that progressive training in sports performance improves the mechanisms of cardiorespiratory integration, which results in the optimization of response to changes in blood oxygen saturation. It seems likely that there are optimal zones of these interactions for each particular kind of sport. These zones can serve to identify athletes with prospects for a high qualification level.

### Limitations

Since these are our first results showing the effect of acute hypoxia on CRC in humans, we cannot conclude definitely whether the associations observed are specific features of the runners or are peculiar to sportsmen of other types. The next limitation is associated with the low time resolution of the measured parameters and the possible transient nature of the investigated phenomena.

### Further Considerations

In the future, we consider it appropriate to confirm these findings in more numerous groups and to investigate the effect of hypoxia on cardiorespiratory coupling in other sports types.

## Conclusion

Highly qualified runners have improved mechanisms of intersystem integration through an increase in the accuracy of cardiorespiratory regulation. In hypoxia, the improvement manifests itself in the strengthening of CRC at frequencies 0.07–0.08 Hz. Strengthening cross-system integration provides optimal responses to hypoxic exposure and reflects the adaptive adjustment of the cardio-respiratory system in athletes during intense aerobic training. Such an increase in coupling between two systems, synergistically working on one function, can serve as an additional sign for the prognosis of qualification level in runners.

## Data Availability Statement

The datasets generated for this study are available on request to the corresponding author.

## Ethics Statement

The studies involving human participants were reviewed and approved by the Ethics Committee of the Scientific Research Institute of Physiology and Basic Medicine. The patients/participants provided their written informed consent to participate in this study.

## Author Contributions

DU and SK conceptualized the research question and study design and supervised the entire project. DU and VB performed the data analysis. All authors interpreted the results. MZ and VG drafted the manuscript. VM edited the English text. VG, VM, NB, and DU critically reviewed and significantly contributed to the manuscript. All authors contributed to the article and approved the submitted version.

## Conflict of Interest

The authors declare that the research was conducted in the absence of any commercial or financial relationships that could be construed as a potential conflict of interest.
